# Dietary insulin index and insulin load in relation to hypertriglyceridemic waist phenotype and low brain derived neurotrophic factor in adults

**DOI:** 10.3389/fnut.2022.980274

**Published:** 2022-09-15

**Authors:** Zahra Hajhashemy, Keyhan Lotfi, Farnaz Shahdadian, Parisa Rouhani, Zahra Heidari, Parvane Saneei

**Affiliations:** ^1^Department of Community Nutrition, Nutrition and Food Security Research Center, School of Nutrition and Food Science, Isfahan University of Medical Sciences, Isfahan, Iran; ^2^Student Research Committee, Isfahan University of Medical Sciences, Isfahan, Iran; ^3^Department of Community Nutrition, School of Nutritional Sciences and Dietetics, Tehran University of Medical Sciences, Tehran, Iran; ^4^Department of Clinical Nutrition, Nutrition and Food Security Research Center, School of Nutrition and Food Science, Isfahan University of Medical Sciences, Isfahan, Iran; ^5^Department of Biostatistics and Epidemiology, School of Health, Isfahan University of Medical Sciences, Isfahan, Iran

**Keywords:** dietary insulin load, brain-derived neurotrophic factor, dietary insulin index, hypertriglyceridemic waist phenotype, cross-sectional study, adults

## Abstract

**Background:**

The evidence about the relation of the insulinemic potential of food with visceral obesity and brain-derived neurotrophic factor (BDNF) was limited. We aimed to investigate the relation of dietary insulin index (DII) and dietary insulin load (DIL) with hypertriglyceridemic waist phenotype (HTGW) and serum BDNF in Iranian adults.

**Methods:**

This cross-sectional study included 528 middle-aged adults (45.6% women), using a multistage cluster random-sampling method. Dietary intakes were assessed using a validated semi-quantitative 168-item food frequency questionnaire. Blood samples were collected after 12 h of fasting for assessing the serum BDNF and triglyceride concentrations. HTGW was defined as triacylglycerol ≥ 150 mg/dL plus enlarged waist circumference. The values less than the first decile of serum BDNF were considered as the low level.

**Results:**

Individuals in the top tertile of DIL, in comparison to those in the bottom tertile, had higher odds of HTGW in both crude (OR = 1.96, 95% CI: 1.14–3.37) and fully adjusted model (OR = 6.10, 95% CI: 1.58–23.53). However, the relation between DII and odds of HTGW was statistically insignificant in crude (OR = 1.30, 95% CI: 0.78–2.16) and maximally adjusted model (OR = 1.25, 95% CI: 0.65–2.40). After considering confounders, participants in the top tertile of DIL had marginally higher odds of having low BDNF values (OR = 2.00, 95% CI: 0.95–4.21). Nevertheless, the association between DII and odds of low BDNF values was statistically insignificant.

**Conclusion:**

This population-based study demonstrated that adults with higher DIL had significantly higher chance of HTGW phenotype and slightly higher chance for low BDNF level. DII was not associated with HTGW phenotype or BDNF values.

## Introduction

Abdominal obesity and visceral obesity became prevalent common public health problems worldwide, due to technological development, sedentary lifestyle, and dietary habits (1, 2). Visceral fat is strongly related to increased risk of non-communicable diseases (NCD) such as dyslipidemia, insulin resistance, type 2 diabetes, chronic inflammation, cardiovascular disease (CVDs), metabolic syndrome (MetS), some cancers, Alzheimer’s disease, and mortality (3–7). Considering that visceral fat accumulation would lead to central adiposity and insulin resistance in adipose tissues, circulating free fatty acids in plasma would be increased and converted to triacylglycerol in the liver (8–11). Therefore, hypertriglyceridemic waist phenotype (HTGW), which is defined as abdominal obesity along with hypertriglyceridemia, is a practical index for predicting visceral fat accumulation (12, 13). Although magnetic resonance imaging and computed tomography (CT) scan are the gold standards for visceral fat measurement, because of applying radioactive rays and high expenses, these tools are not appropriate for epidemiologic studies. Also, waist circumference (WC) values cannot be used to distinguish between subcutaneous and visceral fat; so, it is not a proper tool for visceral obesity assessment (2, 14, 15).

Previous studies documented that brain-derived neurotrophic factor (BDNF), as a member of the neurotrophic factors family which is synthesized in neurons, endothelial cells, immune cells, adipocytes, and monocytes (16–18), plays an important role in regulating the growth, survival, and maintenance of neurons (19). Exercise is supposed to increase BDNF concentration, because the stored BDNF in the brain and platelets is released during the exercise (20). BDNF, as a novel contraction-induced muscle cell-derived protein, can enhance fat oxidation in skeletal muscle (21). In addition, more recent evidences have indicated interactions between serum BDNF and metabolic health status, the balance of energy expenditure, cardiovascular homeostasis, and control of lipid and glucose levels (22–24). It seems that insulin resistance and fat accumulation are related to low levels of serum BDNF (22). Therefore, low serum BDNF level is involved in the pathogenesis of both MetS and neurodegenerative diseases (NDD) like Huntington’s disease, Parkinson’s disease, Alzheimer’s disease, and depression (22).

Regarding the motion pathways, insulin resistance is the key factor in the incidence of visceral obesity and low serum BDNF concentrations. Although there are several risk factors for insulin resistance and visceral fat accumulation, a diet with high insulin index or insulin load could be a main risk factor, because of its insulinemic potential and its direct effect on post-prandial insulin and consequent insulin resistance and visceral adiposity. The relations of several dietary patterns including posteriori-derived dietary patterns (25) and plant-based diet (26) with visceral adiposity have been investigated. Moreover, previous publications investigated the relation of dietary insulin index (DII) and dietary insulin load (DIL) with metabolic disorders such as general obesity (27) and MetS (28). On the other hand, multiple trials have assessed the effect of some dietary patterns such as the Mediterranean diet (29), reduced-calorie diet (30), and carbohydrate-restricted Paleolithic-based diet (31) on serum BDNF; however, the evidence about the relationship between usual long-term dietary intakes with serum BDNF levels in large representative populations was limited. As far as we know, there is no population-based study that investigated the relation of dietary insulin index (DII) and dietary insulin load (DIL) with HTGW or serum BDNF concentrations. Hence, we aimed to investigate these relationships in Iranian adults. The hypothesis of the current study was that DII and DIL were directly associated with odds of HTGW phenotype and low-BDNF level.

## Materials and methods

### Study design and participants

This cross-sectional study was performed on a representative sample of adults in a large central city in Iran in 2021. Considering a prevalence of 17% for HTGW among Iranian adults (32), a confidence of 95%, and precision (d) of 4%, 339 individuals were approximately required for this study. However, considering the high prevalence of COVID-19 pandemic during data collection, a total of 600 eligible participants were invited to participate in the study. A multistage cluster random-sampling method was used to select 600 adults (both gender) aged 20–60 years from 20 schools in Isfahan city. In order to have a representative sample of the general adult population with different socioeconomic statuses, we included all adults who were working in the selected schools, including employees, teachers, school managers, assistants, and crews. However, subjects with the following criteria were not included: (1) being pregnant or lactating; (2) following a special diet; (3) having a prior history of cardiovascular disease, stroke, type 1 diabetes, and cancer. Among invited subjects, 543 of them agreed to participate in our investigation. In addition, we excluded individuals with the following criteria: (1) had left more than 70 items blank on the food frequency questionnaire (*n* = 4); (2) reported a total energy intake outside the range of 800–4,200 kcal/day (as under-reporters and over-reporters of energy intake) (*n* = 3); (3) did not have data of their waist circumference (WC) measurement (*n* = 7); and (4) did not accept blood draw (*n* = 1). Finally, 528 adults were included in the current analysis (response rate: 90.5%). Written informed consent was obtained from each participant. The protocol of the study was ethically approved by the local Ethics Committee of Isfahan University of Medical Sciences in 2021 (no. IR.MUI.RESEARCH.REC.1399.613).

### Assessment of dietary intake

We assessed the usual dietary intake of individuals through a validated Willett-format semi-quantitative 168-item food frequency questionnaire (FFQ) (33). A previous validation study of this FFQ on 132 middle-aged adults revealed reasonable correlations between dietary intakes assessed by FFQ and those obtained from multiple 24-h dietary recalls (33). The correlation coefficients between the dietary intakes obtained from the FFQ and those from the twelve 24-h dietary recalls were 0.55 for total energy, 0.65 for proteins, 0.59 for fat, 0.67 for fiber, and 0.65 for magnesium. The reliability of the FFQ was assessed by comparing nutrient intakes obtained from the FFQ on two occasions 1-year apart. Overall, these data supported that this FFQ could provide reasonably valid measures of the usual dietary intakes among Iranian adults (33). An expert dietitian instructed the study participants to complete the FFQ by reporting the frequency and amount of each food item that they have consumed in the preceding year. Then, the portion sizes of consumed foods were converted to g/day through the use of household measures (34). After that, we entered all food items into Nutritionist IV software, to obtain daily intake of energy and all nutrients.

### Assessment of dietary insulin index and dietary insulin load

We used food insulin index (FII), which refers to the ratio of incremental insulin area under the curve over 2 h in response to the consumption of a 1000-kJ portion of the test food to the area under the curve after ingestion of a 1000-kJ portion of the reference food. The FII for each item was obtained from the previous publications of Holt et al. (35), Bao et al. (36), Bell et al. (37), and Sadeghi et al. (28) that provided a comprehensive list of FIIs. For food items that their FIIs were not reported in these studies, FIIs of similar foods were used.

The insulin load of each food was calculated by the following formula:

Insulin load of a given food = insulin index of that food × amount of that food consumed (g/d) × energy content per 1 g of that food (g/d) (38). DIL for each person was provided through the sum up of the insulin load of all food items consumed in the last year. Then, DII for each participant was computed by dividing DIL by total energy intake.

### Assessment of hypertriglyceridemic waist phenotype and low serum brain-derived neurotrophic factor values

WC was measured to the nearest 0.1 cm through the use of a non-stretchable tape measure. WC was recorded after a normal expiration, by measuring halfway between the lower rib margin and the iliac crest and without any pressure on the body surface. WC measurement of each subject was repeated and the average of two measurements was considered in the analysis. Blood samples were collected after 12 h of fasting; blood samples were allowed to clot, and then were centrifuged to separate serum. Serum triglyceride concentration (TG) was determined by the enzymatic-colorimetric method. The ELISA kits were used to measure serum BDNF values (Zellbio, Veltlinerweg, Germany).

Based on a recently published study that has defined WC cut-off-points in Iranian adults (39), we considered the cut-point of 98 cm for men and 84 cm for women as the threshold for an enlarged WC. In addition, according to the NCEP ATP III, TG ≥ 150 mg/dL was considered as hypertriglyceridemia (40). Based on the mentioned cut-off-points, we categorized participants in to 4 phenotypes including: (1) HTGW (enlarged WC and high triglycerides) [triacylglycerol ≥ 150 mg/dL plus WC ≥ 98 cm (men) and ≥ 84 cm (women)]; (2) enlarged WC and normal triglycerides [triacylglycerol ≤ 150 mg/dL plus WC ≥ 98 cm (men) and ≥ 84 cm (women)]; (3) normal WC and high triglycerides [triacylglycerol ≥ 150 mg/dL plus WC ≤ 98 cm (men) and ≤ 84 cm (women)]; (4) normal WC and normal triglycerides [triacylglycerol ≤ 150 mg/dL plus WC ≤ 98 cm (men) and ≤ 84 cm (women)].

Based on a previous study (29), deciles of serum BDNF concentrations were computed and the bottom decile (D1) (with serum BDNF level of 0.074–0.466 ng/mL or <0.47 ng/mL) was considered as the low serum BDNF level.

### Assessment of other variables

Height and weight were measured while subjects stood with minimal clothing and without shoes. Height was measured to the nearest 0.1 cm through the use of a tape measure. Weight was measured using the body composition analyzer (Tanita MC-780MA, Tokyo, Japan). Weight (kg) divided by the height (m) squared to compute the body mass index (BMI). After 5 min of resting time, blood pressure was measured twice through the use of a digital sphygmomanometer (OMRON, M3, HEM-7154-E, Japan), with an accuracy of 0.5 mmHg, in a sitting position; the mean of two measurements was recorded for each participant.

Data of additional confounders such as age, sex, marital status, education, smoking habits, homeownership, medical history of diseases and medication use were gathered through the use of a self-reported questionnaire. Furthermore, physical activity was measured by the validated International Physical Activity Questionnaires (IPAQ) questionnaire (41). Depression was also assessed using the validated Hospital Anxiety and Depression Scale (HADS) for the Iranian population (42).

### Statistical analysis

The Kolmogorov–Smirnov test was applied to examine the normality of quantitative variables. Mean ± SD/SE and percentage were respectively reported for continuous and categorical variables. First, individuals were distributed in tertiles of DIL and DII. Then, the categorical and continuous variables were compared across tertiles of DIL and DII, by the use of the chi-square test and one-way analysis of variance (ANOVA). Analysis of covariance (ANCOVA) was applied to report age, sex, and energy-adjusted dietary intakes of participants across tertiles of DIL and DII. Using the binary logistic regression, the odds ratio (OR) of HTGW phenotype across tertiles of DIL and DII was calculated, in crude and multivariable-adjusted models. In the first model, the effects of age, sex, and energy intake were controlled. In addition, education, smoking, physical activity, marital status, history of diabetes, hypertension, use of anti-hyperlipidemic medication, family size and homeownership were adjusted in the second model. In the third model, the effect of BMI was additionally controlled. The first tertile of DIL or DII was considered as the reference category in all models. To determine trends, DIL or DII tertiles were treated as continuous variables in logistic regression models. Additionally, crude and multivariable-adjusted models were used to obtain odds of low BDNF values (<0.47 ng/mL) in tertiles of DIL and DII. The effects of age and sex were controlled in the first model. In the second model, depression, hypertension, hyperlipidemia, history of diabetes, and physical activity were adjusted. SPSS software version 20 was used for all statistical analyses. *P*-values less than 0.05 were considered statistically significant.

## Results

The current population-based study was conducted on 528 adults with a mean age of 42.5 (±11.1) years and an average BMI of 26.92 (±4.41) kg/m^2^; 45.6% of the study participants were female. Among study subjects, 21.4% of them had HTGW phenotype (*n* = 113) and the others belonged to the enlarged WC and normal triglycerides (33.9%, *n* = 179), normal WC and high triglycerides (15.1%, *n* = 80) and normal WC and normal triglycerides (29.5%, *n* = 156) phenotypes. The average serum BDNF was 1.20 (ng/mL) among the study population and 0.37 (ng/mL) among subjects with low BDNF levels or those in the first decile of serum BDNF.

General characteristics of study subjects across tertiles of DIL and DII are provided in Table 1. Those in the top tertile of DIL and DII were more likely to be male (*P* < 0.001). We observed significant increasing trends for the weight (*P* < 0.001), height (*P* < 0.001), and waist circumference (*P* = 0.01) across tertiles of DIL. Additionally, there was a significant increasing trend for height (*P* < 0.001) across tertiles of DII. Nevertheless, the distribution of other variables was not significantly different across tertiles of DIL and DII.

**TABLE 1:** General characteristics and cardiometabolic factors of study participants across tertiles of DIL and DII^a^.

	Tertiles of DIL				Tertiles of DII			
		
	T1 (*n* = 171)	T2 (*n* = 178)	T3 (*n* = 179)	*P* ^b^	T1 (*n* = 171)	T2 (*n* = 177)	T3 (*n* = 180)	*P* ^b^
Range	<79808.09	79871.39–107574.00	>107574.00		<40.61	40.63–44.21	>44.21	
Sex, (male) (%)	45.0	51.1	66.5	<0.001	40.9	50.3	71.1	<0.001
Age (year)	42.9 ± 10.8	43.2 ± 11.1	41.5 ± 11.4	0.31	43.9 ± 10.9	42.1 ± 10.3	41.7 ± 12.1	0.14
Weight (kg)	73.6 ± 12.8	74.1 ± 15.8	79.4 ± 14.0	<0.001	74.3 ± 14.5	75.8 ± 15.0	77.1 ± 13.9	0.18
Height (cm)	165 ± 8.6	167 ± 8.1	169 ± 8.4	<0.001	165 ± 8.2	167 ± 9.1	169 ± 7.8	<0.001
BMI^5^ (kg/m^2^)	26.7 ± 4.0	26.4 ± 5.0	27.4 ± 4.0	0.09	27.0 ± 4.7	26.9 ± 4.3	26.8 ± 4.1	0.93
Hip circumference (cm)	104 ± 7.2	104 ± 9.6	105 ± 7.2	0.81	104 ± 8.6	104 ± 8.0	104 ± 7.7	0.63
Waist circumference (cm)	91.1 ± 10.4	91.6 ± 12.1	95.1 ± 11.4	0.01	91.6 ± 11.5	92.5 ± 11.5	93.6 ± 11.3	0.25
TG (mg/dL)	149 ± 2.9	153 ± 2.9	156 ± 3.2	0.33	148 ± 2.8	151 ± 2.8	158 ± 3.4	0.06
BDNF (ng/mL)	1.18 ± 0.03	1.32 ± 0.16	1.11 ± 0.04	0.33	1.15 ± 0.03	1.36 ± 0.16	1.10 ± 0.04	0.15
Physical activity (MET. min/wk)	872 ± 85	828 ± 82	1077 ± 107	0.13	1043 ± 95	787 ± 81	947 ± 100	0.14
Education (University graduated) (%)	88.2	92.1	86.4	0.22	89.9	89.8	87.2	0.66
Marital status (Married) (%)	82.1	83.6	81.5	0.94	79.9	85.8	81.5	0.29
Smoking status (Smokers) (%)	2.0	4.3	3.2	0.27	1.3	5.1	3.0	0.14
Family size (>4) (%)	13.6	14.0	16.5	0.71	15.3	12.6	16.2	0.62
House ownership (yes) (%)	72.0	78.7	74.9	0.42	77.9	77.1	69.0	0.12
Hypertension (yes) (%)	21.3	24.2	28.5	0.29	22.4	22.7	28.9	0.27
History of type 2 diabetes (yes) (%)	5.9	3.9	6.1	0.59	6.5	5.1	4.4	0.68
Antihyperlipidemic drug use (yes) (%)	9.5	5.8	9.7	0.34	10.1	7.1	8.0	0.57

^a^For continuous variables, values are Mean ± SD, except for TG, BDNF and physical activity which are Mean ± SE. For categorical variables, values are percentage. ^b^P-value obtained from one way ANOVA and χ^2^ test for quantitative and categorical variables, respectively. BMI, Body Mass Index; TG, triglycerides; BDNF, brain derived neurotrophic factor.

Dietary intakes of study participants across tertiles of DIL and DII are provided in Table 2. There were significant decreasing trends for intake of protein (*P* = 0.01), cholesterol (*P* < 0.001), saturated fatty acids (SFA) (*P* < 0.001), monounsaturated fatty acids (MUFA) (*P* < 0.001), polyunsaturated fatty acids (PUFA) (*P* < 0.001), vitamin E (*P* = 0.01) and red and processed meat (*P* = 0.01) across tertiles of DIL. Additionally, there were significant increasing trends for intake of energy (*P* < 0.001) and refined grains (*P* < 0.001) in DIL tertiles. Moreover, participants in the top tertile of DII in comparison to the bottom tertile had a significantly lower intake of protein (*P* < 0.001), fat (*P* < 0.001), cholesterol (*P* < 0.001), SFA (*P* < 0.001), MUFA (*P* < 0.001), PUFA (*P* < 0.001), calcium (*P* < 0.001), vitamin E (*P* = 0.02), vegetables (*P* < 0.001), red and processed meat (*P* < 0.001) and dairy (*P* < 0.001). However, subjects in the last tertile of DII had a higher intake of carbohydrates (*P* < 0.001) and refined grains (*P* < 0.001), compared with the first tertile.

**TABLE 2:** Dietary intakes (energy, macro/micro nutrients and food groups) of study participants across tertiles of DIL and DII^a^.

	Tertiles of DIL				Tertiles of DII			
		
	T1 (*n* = 171)	T2 (*n* = 178)	T3 (*n* = 179)	*P* ^b^	T1 (*n* = 171)	T2 (*n* = 177)	T3 (*n* = 180)	*P* ^b^
Range	<79808.09	79871.39–107574.00	>107574.00	–	<40.61	40.63–44.21	>44.21	–
**Nutrient**								
Energy, kcal	1570 ± 26	2205 ± 26	3015 ± 26	<0.001	2315 ± 52	2268 ± 50	2242 ± 51	0.61
Protein,% of energy	14.8 ± 0.2	14.1 ± 0.2	13.8 ± 0.2	0.01	15.1 ± 0.2	14.6 ± 0.2	12.9 ± 0.2	<0.001
Carbohydrate,% of energy	60.0 ± 0.6	60.6 ± 0.6	61.9 ± 0.6	0.08	55.6 ± 0.5	60.3 ± 0.5	66.3 ± 0.5	<0.001
Fat,% of energy	26.9 ± 0.5	27.1 ± 0.5	26.3 ± 0.5	0.45	31.2 ± 0.4	26.8 ± 0.4	22.5 ± 0.4	<0.001
Cholesterol, mg	337 ± 13	286 ± 8	209 ± 13	<0.001	321 ± 8	290 ± 8	220 ± 8	<0.001
SFA, gr	26.9 ± 0.8	23.3 ± 0.5	16.8 ± 0.8	<0.001	25.7 ± 0.5	22.9 ± 0.5	18.4 ± 0.5	<0.001
MUFA, gr	27.0 ± 0.7	22.4 ± 0.4	15.9 ± 0.7	<0.001	25.4 ± 0.4	21.9 ± 0.4	17.9 ± 0.4	<0.001
PUFA, gr	20.1 ± 0.8	16.9 ± 0.5	11.1 ± 0.8	<0.001	19.6 ± 0.5	15.4 ± 0.5	13.2 ± 0.5	<0.001
Fructose, gr	21.8 ± 1.3	20.9 ± 0.8	20.4 ± 1.4	0.80	20.3 ± 0.9	20.5 ± 0.8	22.3 ± 0.8	0.25
Calcium, mg	955 ± 43	909 ± 28	908 ± 44	0.64	999 ± 28	949 ± 27	826 ± 27	<0.001
Vitamin E, mg	7.93 ± 0.35	6.73 ± 0.23	5.98 ± 0.36	0.01	7.29 ± 0.24	6.96 ± 0.23	6.37 ± 0.23	0.02
Total fiber, gr	21.9 ± 0.7	21.0 ± 0.4	20.4 ± 0.7	0.49	21.6 ± 0.4	21.0 ± 0.4	20.7 ± 0.4	0.43
**Food groups**								
Whole grains, g/d	79.0 ± 9.1	96.6 ± 5.9	109 ± 9.3	0.16	94.7 ± 6.1	97.9 ± 5.9	93.1 ± 6.0	0.84
Refined grains, g/d	192 ± 18	262 ± 11	361 ± 18	<0.001	214 ± 12	273 ± 11	329 ± 11	<0.001
Fruits, g/d	605 ± 37	555 ± 23	507 ± 38	0.33	541 ± 24	549 ± 23	574 ± 24	0.62
Vegetables, g/d	379 ± 25	339 ± 16	281 ± 26	0.10	387 ± 16	329 ± 16	283 ± 16	<0.001
Red and processed meat, g/d	79.5 ± 5.2	71.0 ± 3.3	53.4 ± 5.3	0.01	71.8 ± 3.4	75.2 ± 3.3	56.7 ± 3.3	<0.001
Dairy, g/d	338 ± 30	306 ± 19	307 ± 31	0.65	368 ± 20	330 ± 19	254 ± 19	<0.001
Nuts, soy and legumes, g/d	52.1 ± 4.4	51.2 ± 2.8	49.8 ± 4.5	0.96	53.7 ± 2.9	50.5 ± 2.8	49.0 ± 2.8	0.50

^a^Values are Mean ± SE. Energy intake and macronutrients were adjusted for age and gender; all other values were adjusted for age, gender and energy intake. ^b^P-value obtained from ANCOVA test for adjustment of energy intake. SFA, Saturated fatty acids; MUFA, Monounsaturated fatty acids; PUFA, Polyunsaturated fatty acids.

**FIGURE 1:**
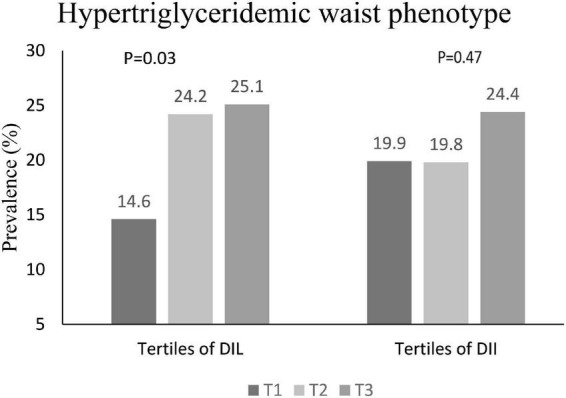
Prevalence of HTGW phenotype in tertiles of DIL and DII.

The prevalence of HTGW in tertiles of DIL and DII is shown in Figure 1. In the first, second and third tertile DIL, 14.6, 24.2, and 25.1% of subjects respectively had HTGW phenotype; this increasing trend was statistically significant (*P* = 0.03). However, the prevalence of HTGW phenotype was not significantly different across tertiles of DII (*P* = 0.47).

**FIGURE 2:**
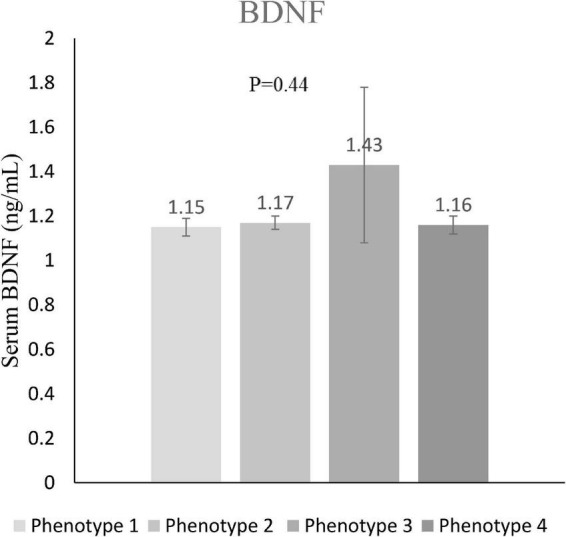
Serum BDNF values in different phenotypes of serum triacylglycerol concentration and waist circumference. Value of serum BDNF in phenotype 1 (enlarged waist circumference and high triglyceride concentrations) was 1.15 ± 0.04 (mean ± SE); in phenotype 2 (enlarged waist circumference and normal triglycerides) was 1.17 ± 0.03; in phenotype 3 (normal waist circumference and high triglyceride concentrations) was 1.43 ± 0.35; and in phenotype 4 (normal waist circumference and normal concentrations) was 1.16 ± 0.04.

The distribution of four different phenotypes based on serum triacylglycerol concentration and waist circumference across tertiles of DIL and DII is presented in Table 3. Although the prevalence of these phenotypes was slightly different in tertiles of DIL (*P* = 0.06), there was no significant difference in tertiles of DII (*P* = 0.65). Moreover, the prevalence of the second phenotype (enlarged waist circumference and normal triglyceride values) was higher than three other phenotypes across tertiles of both DIL and DII. The mean values of serum BDNF in four different phenotypes based on serum triacylglycerol concentration and waist circumference values are reported in Figure 2. Participants with the HTGW phenotype had the lowest serum BDNF values [1.15 ± 0.04 (SE)] and those with the third phenotype (normal WC and high triglyceride values) had the highest levels of serum BDNF [1.43 ± 0.35 (SE)]; however, these differences were not statistically significant (*P* = 0.44).

**TABLE 3:** Distribution of different phenotypes of serum triacylglycerol concentration and waist circumference (WC) across tertiles of DIL and DII.

	Tertiles of DIL				Tertiles of DII			
		
	T1 (*n* = 171)	T2 (*n* = 178)	T3 (*n* = 179)	*P* ^a^	T1 (*n* = 171)	T2 (*n* = 177)	T3 (*n* = 180)	*P* ^a^
Range	<79808.09	79871.39–107574.00	>107574.00		<40.61	40.63–44.21	>44.21	
Phenotypes				0.06				0.65
Enlarged WC and high triglycerides^b^ (%)	14.6	24.2	25.1		19.9	19.8	24.4	
Enlarged WC and normal triglycerides^c^ (%)	40.4	27.5	34.1		37.4	35.6	28.9	
Normal WC and high triglycerides^d^ (%)	17.0	14.6	14.0		13.5	14.7	17.2	
Normal WC and normal triglycerides^e^ (%)	28.1	33.7	26.8		29.2	29.9	29.4	

^a^For differences among tertiles of DIL and DII (chi-square test). ^b^Triacylglycerol ≥ 150 mg/dL plus WC ≥ 98 cm (men) and WC ≥ 84 cm (women). ^c^Triacylglycerol ≤ 150 mg/dL plus WC ≥ 98 cm (men) and WC ≥ 84 cm (women). ^d^Triacylglycerol ≥ 150 mg/dL plus WC ≤ 98 cm (men) and WC ≤ 84 cm (women). ^e^Triacylglycerol ≤ 150 mg/dL plus WC ≤ 98 cm (men) and WC ≤ 84 cm (women).

Multivariate adjusted odds ratio (OR) and 95% confidence interval (CI) for HTGW phenotype across tertiles of DIL and DII are presented in Table 4. Subjects in the highest tertile of DIL, in comparison to those in the lowest tertile, had 96% higher odds of HTGW (OR = 1.96, 95% CI: 1.14–3.37) in the crude model. After adjustment for potential confounders, this association became stronger; such that individuals in the third tertile of DIL had 6.10 times higher odds of HTGW, compared to those in the first tertile (OR = 6.10, 95% CI: 1.58–23.53). Additionally, there was a significant increasing trend for the odds of HTGW across tertiles of DIL (P_trend_ = 0.01). Nevertheless, there was no significant association between DII and HTGW, in the crude model (OR = 1.30, 95% CI: 0.78–2.16). After controlling potential confounders, this relation did not change (OR = 1.25, 95% CI: 0.65–2.40). Moreover, there was no significant trend for the prevalence of HTGW across tertiles of DII (P_trend_ = 0.46).

**TABLE 4:** Multivariate adjusted odds ratio (OR) and 95% confidence interval (CI) for HTGW phenotype across tertiles of DIL and DII^a^.

	Tertiles of DIL				Tertiles of DII			
		
	T1 (*n* = 171)	T2 (*n* = 178)	T3 (*n* = 179)	*P*-trend	T1 (*n* = 171)	T2 (*n* = 177)	T3 (*n* = 180)	*P*-trend
Cases	25	43	45		34	35	44	
Crude	1.00 (Ref)	1.86 (1.07, 3.21)	1.96 (1.14, 3.37)	0.02	1.00 (Ref)	0.99 (0.58, 1.68)	1.30 (0.78, 2.16)	0.29
Model 1	1.00 (Ref)	2.40 (1.22, 4.69)	3.61 (1.29, 10.13)	0.01	1.00 (Ref)	1.04 (0.61,1.78)	1.43 (0.84, 2.43)	0.18
Model 2	1.00 (Ref)	2.82 (1.26, 6.34)	3.94 (1.16, 13.35)	0.03	1.00 (Ref)	0.72 (0.38, 1.35)	1.17 (0.64, 2.16)	0.56
Model 3	1.00 (Ref)	4.30 (1.73, 10.69)	6.10 (1.58, 23.53)	0.01	1.00 (Ref)	0.67 (0.34, 1.34)	1.25 (0.65, 2.40)	0.47

^a^All values are odds ratios and 95% confidence intervals. P-trend was obtained by the use of DIL or DII tertiles as a continuous rather than categorical variable. Model 1, Adjusted for age, sex, and energy intake; Model 2, Additionally, adjusted for marital status, education, family size, smoking, hypertension, diabetes, physical activity and homeownership; Model 3, Additionally, adjusted for body mass index (BMI).

Multivariate adjusted odds ratio (OR) and 95% confidence interval (CI) for the prevalence of low BDNF values (<0.47 ng/mL) across tertiles of DIL and DII are provided in Table 5. Although the relation between DIL and odds of low BDNF values was statistically insignificant in the crude model (OR = 1.79, 95% CI: 0.87–3.66), after taking potential confounders into account, this relation became marginally significant; such that participants in the top tertile of DIL had marginally 2 times higher odds for low BDNF values (OR = 2.00, 95% CI: 0.95–4.21). Additionally, there was a marginally significant increasing trend for the odds of low BDNF values across tertiles of DIL (*P* = 0.07). Nevertheless, the association between DII and the prevalence of low BDNF values was statistically insignificant (OR = 1.42, 95% CI: 0.72–2.79), even after adjustment for potential confounders (OR = 1.49, 95% CI: 0.73–3.06). Also, no significant trend was found for low BDNF prevalence across DIL categories (*P* = 0.25).

**TABLE 5:** Multivariate adjusted odds ratio (OR) and 95% confidence interval (CI) for very low BDNF (<0.47 ng/mL, 1st decile) across tertiles of DIL and DII^a^.

	Tertiles of DIL				Tertiles of DII			
	T1 (*n* = 171)	T2 (*n* = 178)	T3 (*n* = 179)	*P*-trend	T1 (*n* = 171)	T2 (*n* = 177)	T3 (*n* = 180)	*P*-trend
**Cases**	13	18	23		16	15	23	
Crude	1.00 (Ref)	1.36 (0.64, 2.88)	1.79 (0.87, 3.66)	0.11	1.00 (Ref)	0.89 (0.43, 1.87)	1.42 (0.72, 2.79)	0.29
Model 1	1.00 (Ref)	1.39 (0.66, 2.95)	1.92 (0.92, 3.98)	0.08	1.00 (Ref)	0.92 (0.44, 1.93)	1.57 (0.77, 3.18)	0.24
Model 2	1.00 (Ref)	1.42 (0.66, 3.03)	2.00 (0.95, 4.21)	0.07	1.00 (Ref)	0.89 (0.42, 1.89)	1.49 (0.73, 3.06)	0.26

^a^All values are odds ratios and 95% confidence intervals. P-trend was obtained by the use of DIL or DII tertiles as a continuous rather than categorical variable. Model 1, Adjusted for age and sex; Model 2, Additionally, adjusted for depression, hypertension, hyperlipidemia, history of diabetes, and physical activity.

## Discussion

The current epidemiologic investigation illustrated that more adherence to a diet with higher DIL was related to elevated odds of HTGW phenotype in Iranian adults. Moreover, subjects who followed a diet with higher DIL had slightly higher odds for low BDNF values. Nevertheless, there was no significant relation between DII and HTGW and serum BDNF concentrations.

The present population-based study indicated that a considerable percentage of Iranian adults (21.4%) had HTGW phenotype. This high-risk phenotype was more prevalent among participants with high adherence to a diet with high DIL and those with low serum BDNF values. Considering the role of visceral fat in metabolic disorders and the involvement of BDNF in metabolic health status, identification and management of HTGW and low serum BDNF values are crucial steps in preventing and decreasing the incidence rate of these disorders. Our findings have suggested that clinicians would advise people to adhere to a diet with lower insulinemic potential, in order to prevent these conditions.

Similar to our study, some recently published studies have investigated the relation of DII and DIL with metabolic disorders. Anjom-Shoae et al. investigated 8,691 Iranian middle-aged adults and found no significant association between DIL and general obesity, in contrast to the significant association between DIL and HTGW that was found in the present study. However, they found a straight relationship between DII and general obesity in females, while no significant association was observed in males (27). Moreover, Sadeghi et al. performed a cross-sectional analysis on 5,954 Iranian adults from the Shahedieh cohort study to investigate the relation of DIL and DII with metabolic syndrome. They reported that women in the highest quartile of DII and DIL in comparison to women in the lowest quartile had greater odds of MetS. Among male participants, the third quartile of DIL, compared with the first quartile, was significantly associated with higher odds of MetS (28); the last quartile of DIL as well as categories of DII were not related to MetS in men. Another cross-sectional study on 850 Iranian adults reported no significant association between DII and DIL and obesity, MetS or its components (43). Moreover, the data analysis of Nurses’ Health Study and the Health Professionals Follow-Up Study revealed significant associations only between DII/DIL and two components of MetS (high-TG and low-HDL) (38). These observed differences might be due to tools applied for dietary assessment, the different age ranges of study participants, different variables considered as confounders, and various methods used to compute dietary insulin index and insulin load.

Several previous interventional studies indicated that dietary intakes could have important effects on circulating BDNF levels. The PREDIMED-NAVARRA randomized trial on 243 adults with depression has documented that the Mediterranean diet with virgin olive oil or nuts after 3 years of follow-up could slightly increase circulating BDNF concentrations (29). Another trial was conducted on 17 overweight and obese subjects to determine the effect of a reduced-calorie diet (RCD) on BDNF values. After 3-month adherence to this RCD, a significant increase in BDNF level was found and favorable changes in anthropometric and glycemic indices have occurred (30). Similarly, a cross-over randomized controlled trial with two 4-week phases and a 4-week washout period on 12 adults with MetS has indicated favorable changes in serum BDNF levels after following a carbohydrate-restricted Paleolithic-based diet (CRPD) with less than 50 g carbohydrates per day (31). These favorable changes were observed in both intervention groups with sedentary activity and high-intensity interval training (31). Although multiple clinical trials have investigated the effects of diet and physical activity interventions on serum BDNF levels, the relationship between usual dietary intakes of the general population with serum BDNF values was less studied so far. However, our population-based epidemiologic study on adults indicated that low serum BDNF values were marginally more prevalent among participants who followed a diet with higher DIL.

Some mechanisms have been suggested to explain the relationship between DIL and HTGW phenotype and serum BDNF levels. Adherence to a diet with higher insulinemic potential would lead to higher insulin secretion, carbohydrate oxidation and lower fat oxidation; therefore, following such a diet would promote fat storage, in particular in the abdominal area and increase the risk of visceral obesity (44). A diet with high insulinemic potential would also have a rapid process of digestion, absorption and transformation to glucose; so, such a diet would rapidly increase blood glucose and insulin and with a short interval decrease blood glucose (45). Blood glucose fluctuation would decrease satiety and elevate hunger sensation and calorie intake and consequently would increase the risk of obesity (45, 46). Additionally, higher DII and DIL are related to a higher risk of insulin resistance (47) and metabolic syndrome (28). Oxidative and nitrosative stresses due to elevated post-prandial glucose and visceral obesity would lead to decreased serum BDNF levels (48). Additionally, insulin resistance and raised post-prandial glucose would directly inhibit the BDNF secretion (49). Moreover, experimental studies suggested that the expression of BDNF mRNA was decreased because of stress and high levels of proinflammatory cytokines in the hippocampus (50). Therefore, due to the negative effect of insulin resistance and other mentioned metabolic disorders on serum BDNF levels (22), DII and DIL would be inversely related to serum BDNF levels.

BDNF serves as a neurotransmitter modulator and an anorexigenic factor that is related to the melanocortin-4 receptor gene, dopaminergic system and serotonin signaling (19, 22, 51, 52). Serotonin, which is decreased in depressed patients, is involved in psychological health, feeling, motivation, learning, appetite and sleep (22). Additionally, BDNF is involved in energy homeostasis through the secretion and function of pre-inflammatory cytokine, ghrelin, leptin, insulin and peptide neurotransmitters (53, 54). Therefore, low serum BDNF is related to both neurodegenerative diseases (NDD) such as Huntington’s, Parkinson’s, and Alzheimer’s diseases, depression and metabolic disorders such as diabetes, obesity, dyslipidemia, inflammation and hypertension (22).

The current study has some strengths and weaknesses. We investigated the relation of DIL and DII with HTGW and serum BDNF levels for the first time among Iranian adults. Additionally, the effect of potential confounders was taken into account. Furthermore, the study sample was selected by the use of a multistage cluster random sampling method. Therefore, our population could be representative of the general adult population and the findings could be generalizable to the whole Iranian population. Nevertheless, some limitations should be considered, while interpreting these findings. Due to the cross-sectional design of the study, causality cannot be inferred; more prospective studies are required to find a causal relationship. Although dietary intakes were assessed by the use of a validated FFQ, recall bias along with other potential reporting biases were inevitable and might influence our findings. The range of DII among tertiles was too narrow (T3 vs. T1: 47.4 vs. 37.9), which made it difficult to find the associations with outcomes of interest. DII and DIL have some other restrictions that should be kept in mind while interpreting our findings. DII and DIL are somehow questionable parameters for describing food quality, because these indices cannot discriminate healthy from unhealthy fats. Additionally, fructose intake which is associated with the pathogenesis of metabolic diseases such as non-alcoholic fatty liver disease (NAFLD), obesity, hyperuricemia and hypertension, seems not to stimulate insulin secretion (55). Therefore, fructose rich foods would be considered healthy for being low-insulinogenic. Nevertheless, in the current study, there was no significant difference in fructose intake across tertiles of DII and DIL; therefore, our results might not be influenced by fructose intake. Moreover, besides DII and DIL, the energy and macronutrient intakes as well as energy density of foods, which are more dependent factors to the fat content of foods, would influence the results (56). Such that, in some previous studies on DII and DIL, different energy and macronutrient intakes across categories of DII or DIL might result in significant findings (28). However, in some other investigations with different energy and macronutrient intakes across categories of DII or DIL, no significant findings were found (38, 43). In the current study, we found a significant inverse relation between DIL and HTGW phenotype; while significant differences were found only in case of energy and protein intakes across tertiles of DIL; fat or carbohydrates were not different across DIL categories. Further investigations are needed to determine whether overall observed effects would be explained by energy or carbohydrate intake, irrespective of putative differential insulinemia, or not.

To conclude, this cross-sectional population-based study demonstrated that participants with higher DIL had significantly higher chance of HTGW phenotype. Additionally, subjects who followed a diet with higher DIL had slightly higher chance for low BDNF values. However, more prospective investigations should be conducted to confirm these findings.

## Data availability statement

The raw data supporting the conclusions of this article will be made available by the authors, without undue reservation.

## Ethics statement

The study protocol was approved by the Local Ethics Committee of Isfahan University of Medical Sciences in 2021 (no. IR.MUI.RESEARCH.REC.1399.613). The patients/participants provided their written informed consent to participate in this study.

## Author contributions

ZHA, KL, FS, PR, ZHE, and PS contributed in conception, design, data collection, data interpretation, manuscript drafting, approval of the final version of the manuscript, and agreed for all aspects of the work. All authors contributed to the article and approved the submitted version.

## Abbreviations

FFQ: food frequency questionnaire
OR: odds ratios
95% CI: 95% confidence interval
BMI: body mass index
MUFA: mono unsaturated fatty acids
PUFA: poly unsaturated fatty acids
SFA: saturated fatty acids
TG: triglycerides
ANOVA: analysis of variance
ANCOVA: analysis of covariance
SPSS: statistical package for the social sciences
SD: standard deviation
SE: standard error
FII: food insulin index
MetS: metabolic syndrome
CVD: cardiovascular disease
NCD: non-communicable diseases
CT: computed tomography
HTGW: hypertriglyceridemic waist phenotype
BDNF: brain-derived neurotrophic factor
NDD: neurodegenerative diseases
DII: dietary insulin index
DIL: dietary insulin load
WC: waist circumference
RCD: reduced-calorie diet
CRPD: carbohydrate-restricted Paleolithic-based diet.
